# Utilizing Community Health Needs Assessments (CHNAs) in Nonprofit Hospitals to Guide Population-Centered Outcomes Research for Pediatric Patients: New Recommendations for CHNA Reporting

**DOI:** 10.1089/pop.2018.0049

**Published:** 2019-02-01

**Authors:** Joshua B. Gruber, Weize Wang, Alexandra Quittner, Daria Salyakina, Jennifer McCafferty-Fernandez

**Affiliations:** Research Analytics and Data, Nicklaus Children's Hospital, Miami, Florida.

**Keywords:** pediatric health needs, PCORI, CHNA

## Abstract

Currently, Community Health Needs Assessment (CHNA) reports lack a standard structure, making it difficult to derive meaningful information. However, they have the potential to be a useful tool for analyzing pediatric outcomes, guiding resource allocation, and linking to Patient-Centered Outcomes Research Institute priorities. The objective was to evaluate the utility of CHNA for informing future pediatric, patient-centered outcomes research. The authors analyzed CHNA documents, published before July 1, 2016 by 61 nonprofit hospitals, focusing on 4 metropolitan areas in Florida: Miami, Orlando, Tampa, and Jacksonville. Out of 18 health priorities identified, access to care and obesity were universally recognized as the most urgent pediatric health needs across all hospital types and metropolitan regions. This analysis also yielded insights into key regional differences. The authors advocate that a major change in the CHNA format be implemented using a common set of domains to produce meaningful, interpretable, and comparable results that inform and guide patient-centered health outcomes research.

## Introduction

As a result of Section 9007 of the Affordable Care Act (ACA), all nonprofit hospitals are required to conduct a comprehensive community health needs assessment (CHNA) every 3 years.^1,2^ The goal of the CHNA is to improve community health and wellness by connecting research, resources, and people, while complying with Internal Revenue Service guidelines. Ideally, a CHNA allows hospitals and other health care organizations to identify community health needs and existing gaps in services, strengthen relationships with community partners to improve local health care delivery, and share this information with the public. According to the regulation, CHNAs must be publically available and the hospital must adopt an “implementation strategy” to meet the community health needs identified.

The optimal CHNA process attempts to align several different resources to achieve maximum community benefits.^3^ Initially, hospitals gather both qualitative and quantitative health data on their surrounding community. Qualitative data (eg, perceptions of health priorities, health care barriers) are obtained from surveying and interviewing community members, health officials, physicians, and other key stakeholders. Quantitative data are gathered from secondary resources, such as local health departments, National Institutes of Health, Centers for Disease Control and Prevention, Census Bureau, and other large databases. Information obtained from these resources (eg, prevalence data, socioeconomic indicators) is prioritized and matched with a hospital's strengths and capacity to determine the areas that can be impacted most significantly by applying public health resources.^3^

In theory, a CHNA can help identify and prioritize the health needs of the local pediatric population. The concept of providing an evaluation of a community's current state of health is a critically important one. If conducted correctly, it can facilitate a better understanding of the hospital's role as a health care provider in the community and identify areas for improvement. The CHNA also provides these organizations with objective measures of success (eg, changes in mortality) that can be assessed over time. These measures foster goal setting, policy making, and improvements to procedures and protocols when needed. Similarly, the public can view and track the health of their community and hold hospitals accountable for substandard care or lack of progress on stated goals. A major aim of this study is to evaluate how the CHNAs, collated from 4 metropolitan regions in Florida, can be used to identify critical pediatric health needs across these communities.

Another major benefit of CHNA reports is the potential link to the Patient-Centered Outcomes Research Institute (PCORI). PCORI is a nonprofit organization that funds clinical effectiveness research to achieve better health outcomes in areas most important to *patients*, using valid and standardized methodologies.^4^ CHNA reports can identify and elucidate information gaps and areas for future research that align with topics prioritized by PCORI. This linkage between CHNA reports and PCORI goals would provide a unique opportunity to improve quality of care. The secondary aim of this study was to describe this relationship.

Despite the substantial resources (money, personnel, time,) allocated to create CHNA reports, it is not clear whether they are useful for improving public health, particularly in pediatric populations. CHNAs are a recent requirement of nonprofit hospitals and there is a dearth of published literature on their findings. To date, no studies have examined their ability to provide useful data that guide implementation of future public health initiatives for specific populations. Factors that limit their utility include: the wide range of methodologies used for data collection and prioritization of health needs, variability in the depth of analysis that is possible, and lack of specificity on best practices for utilizing and implementing the results.^5–9^ Therefore, the primary purpose of this study was to assess the feasibility of analyzing CHNA reports to identify pediatric health priorities, using Florida hospitals as an example. This study also explored linkages between CHNAs and PCORI goals, with recommendations to better standardize the CHNA process.

## Methods

**FIG. 1.**
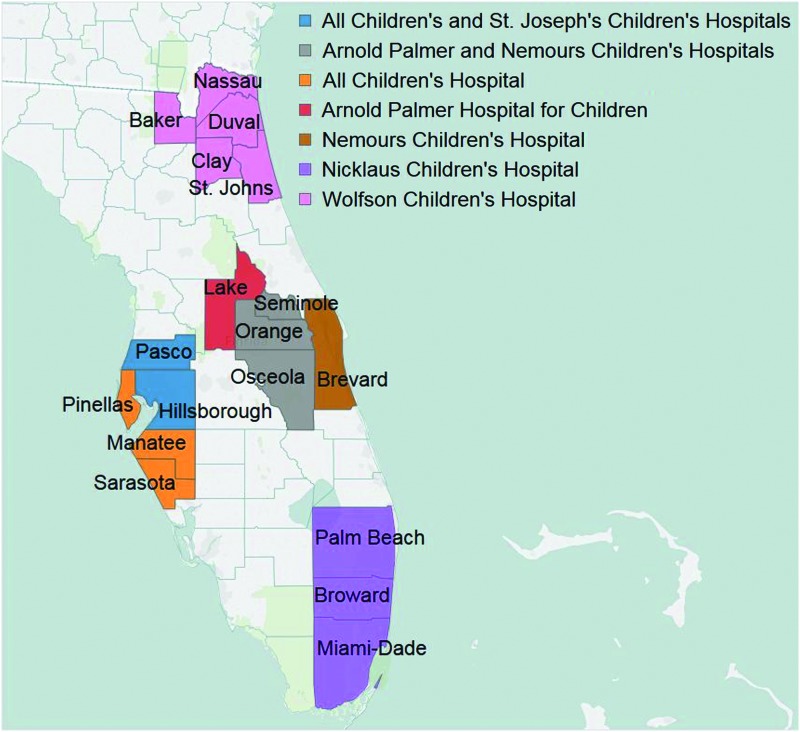
Counties served by freestanding children's hospitals in Florida. Color images are available online.

To evaluate the current health needs of Florida's pediatric patients, CHNA reports were examined for all nonprofit hospitals providing pediatric care, including municipal hospitals. There are 7 freestanding children's hospitals across Florida that exclusively provide pediatric care; the remainder also treat adult and geriatric patients. These 7 hospitals are located in the 4 largest metropolitan areas: Tampa, Jacksonville, Miami, and Orlando (Figure 1). Therefore, this study focused on CHNA reports from the hospitals in these areas. To be included, hospitals had to provide, at minimum, emergency services to pediatric patients. CHNA methodologies, data collection sources, and hospital demographics were evaluated. The latest CHNA report on each hospital's website, published before July 1, 2016, was included in the evaluation.

Of the 61 hospitals providing pediatric services with an available CHNA report, 42 (69%) were private nonprofit, 12 (20%) were publicly or government-owned, and 7 (11%) were independent, freestanding children's hospitals (Supplementary Table S1). The Miami metropolitan region had the largest number of health care facilities (21), followed by Orlando (17), Tampa (12) and Jacksonville (11).

CHNA health priorities were tabulated for each report and compared across hospitals. Although some hospitals submitted individual CHNA reports, a majority (85%) combined their CHNA reports into 1 document to reflect their community partnerships or hospital networks. There were 3 major community health partnerships (ie, Jacksonville, Central Florida, St. Joseph's) filing 3 individual reports, which together included 37 out of the 61 hospitals (Supplementary Table S1). To account for these joint reports, health priorities were counted once for each hospital covered within the CHNA.

Following the tabulation of health priorities, hospitals were categorized into 3 groups: (1) freestanding (independent) children's hospitals (n = 7), (2) hospitals caring for all age groups, with pediatric data separated in the CHNA document (n = 33), and (3) hospitals that provided care to all age groups, but did not separate pediatric health priorities in the document (n = 21). The rationale underlying this categorization was the hypothesis that differences in hospital demographics directly impact the health needs and priorities listed in the CHNA.

Descriptions and definitions of health priorities across CHNA documents were not uniform. Some reports were more detailed than others — listing specific diagnoses (eg, asthma) rather than broad categories (eg, respiratory conditions). Therefore, medically related health needs were grouped together. For example, nutrition, exercise, and obesity were combined. Similarly, all terms related to drugs, alcohol, smoking, or addiction were categorized as “substance use disorder.” Finally, “access to care” was defined as any barrier to health care services related to insurance or cost, physician availability, distance to care, or transportation.

Health needs were compared between hospitals that included distinct pediatric priorities in their CHNA, hospitals that did not, and freestanding children's hospitals. A “priority score” was assigned to each health need for each metropolitan area, ranging from 1 to 18 (18 being the highest priority). The score was based on the number of hospitals that mentioned each priority in their CHNA report. These scores were compared against a weighted mean score for all health priorities. The study team also examined regional differences by documenting how hospitals within each metropolitan area prioritized health needs.

## Results

Initially, a descriptive analysis of freestanding hospitals was conducted highlighting the types of data sources used in their CHNA report. Seven hospitals were in this category, all located within the 4 largest metropolitan areas mentioned (Table 1). Of these hospitals, Nicklaus Children's was the largest with 289 beds, followed by All Children's, and Wolfson Children's. The smallest was Shriner's Children's Hospital (Table 1). Data sources used for the CHNA reports by each freestanding children's hospital were reviewed. As primary sources, most hospitals used a combination of surveys, focus groups, and individual interviews to gain greater insight into the populations they served (eg, children's health, experience of care, nutrition, exercise). Great differences were found in the number of individuals who participated; for example, Nicklaus Children's Hospital accrued 1129 more survey participants for their CHNA document than Arnold Palmer Hospital for Children (Table 1).

**Table 1.** Florida's Freestanding Children's Hospitals

	*All Children's Hospital Johns Hopkins Medicine*	*Arnold Palmer Hospital for Children*	*Nemours Children's Hospital*	*Nicklaus Children's Hospital*	*Shriners Hospitals for Children-Tampa*	*St. Joseph's Children's Hospital*	*Wolfson Children's Hospital*
City	St. Petersburg	Orlando	Orlando	Miami	Tampa	Tampa	Jacksonville
Number of Beds	259	158	95	289	25	202	216
Year of CHNA report	2013	2013	2013	2015	2015	2013	2012
Type of Quantitative Data Sources	Secondary	Secondary	Primary and Secondary	Primary and Secondary	Primary and Secondary	Primary and Secondary	Primary and Secondary
Number of Secondary Data Sources	4	22	8	13	2	9	9
Type of Qualitative Data Source (sample size)	Survey, Focus Groups, Interviews (80)	Survey, Interviews (72)	Survey, Focus Groups (1038)	Survey, Focus Groups, Interviews (1201)	Survey (85)	Focus Groups, Interviews (N/A)	Survey, Focus Groups, Interviews (970)

CHNA, Community Health Needs Assessment.

**FIG. 2.**
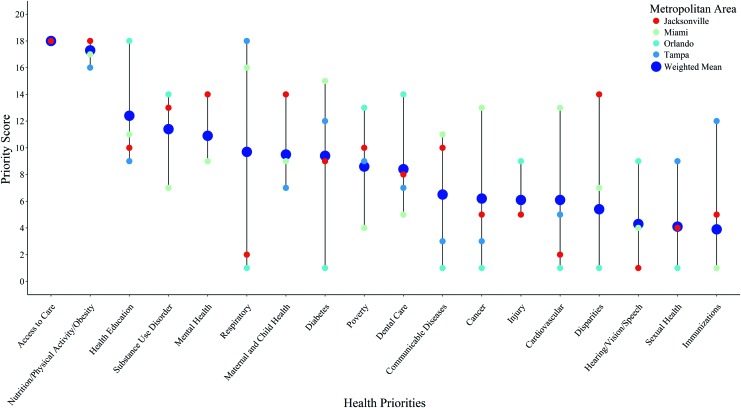
Priority scores for each of the pediatric health needs by the 4 metropolitan regions compared to the weighted mean from 61 hospitals in Florida. A higher score indicates that more hospitals in the metropolitan area placed a priority on that specific health need. The weighted score was calculated by adjusting the total number of hospitals within each of the 4 metropolitan regions (ie, Tampa, Orlando, Jacksonville, Miami). Color images are available online.

**FIG. 3.**
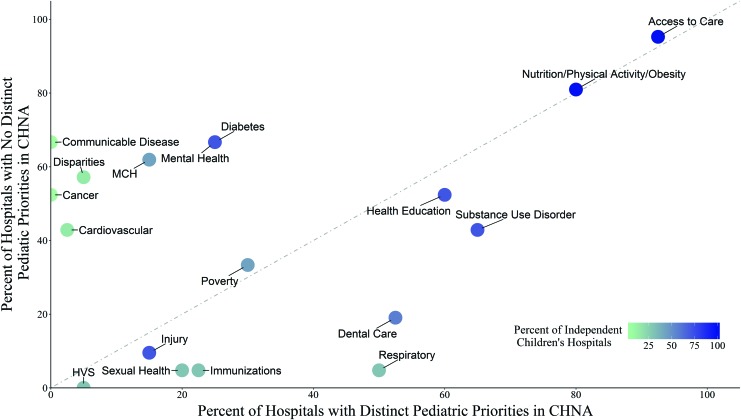
Pediatric health needs by 3 types of hospitals in Florida. The figure shows the proportion of hospitals that included specific health priorities in their Community Health Needs Assessment reports. The 3 types of hospitals included: (1) hospitals with distinct pediatric priorities (x-axis); (2) hospitals with no distinct priorities (y-axis); (3) freestanding children's hospitals (color of the bubble). CHNA, Community Health Needs Assessment; HVS, hearing/vision/speech; MCH, maternal and child health. Color images are available online.

Next, the study team analyzed how these Florida hospitals prioritized pediatric health needs. Community health priorities were grouped in 18 categories (Figure 2). The most frequently mentioned health priority was access to care (57 of 61) followed by nutrition/exercise/obesity (n = 49), mental health (n = 35), and health education (n = 35). The least frequently mentioned health needs were hearing/vision/speech (n = 2), injury (n = 8), and sexual health (n = 9). Among hospitals that separated their pediatric priorities within their CHNA reports, access to care, nutrition/exercise/obesity, and substance use disorders were mentioned most frequently. Among hospitals that did not separate pediatric priorities, access to care, nutrition/exercise/obesity, diabetes, mental health, and communicable diseases were identified as the greatest concerns. Independent children's hospitals mentioned injury, mental health, health education, and substance use disorder most often in their CHNA reports (Figure 3).

Among the 18 health needs identified, access to care was the only one universally prioritized across the 4 metropolitan areas. The second most frequently mentioned priority was nutrition/exercise/obesity. However, none of the other priorities was ranked consistently and there were large discrepancies in how these regions ranked diagnoses and conditions such as respiratory diseases, diabetes, cancer, poverty, disparities, communicable diseases, and immunizations. Unlike other areas, hospitals in the Orlando region placed high importance on improving health education, while hospitals in the Miami region mentioned respiratory conditions, diabetes, cancer, and cardiovascular health more frequently. Only the Tampa region prioritized immunization needs for pediatric patients.

As an exploratory aim, the study team analyzed the linkages between CHNA principles^8^ and PCORI goals^4^ (Table 2). This comparison revealed significant similarities: collaboration, community engagement, evidence-based research, and identifying key patient-centered health needs. The analyses of the CHNA data identified 2 important linkages to PCORI goals. First, PCORI explicitly required data on the outcomes and needs most important to patients. CHNA results consistently identified access to care and nutrition/exercise/obesity as the top 2 health priorities. The second explicit linkage to PCORI was the inclusion of patients as key stakeholders. For example, Nicklaus Children's Hospital obtained 1201 surveys from primary caregivers and key community stakeholders to identify the top pediatric health needs. Thus, patient–caregiver priorities were strongly reflected in the CHNA results.

**Table 2.** Comparison of Community Health Needs Assessment Principles and Patient-Centered Outcomes Research Insitute Goals

*CHNA principles*		*PCORI goals*	
1)	Multi-sector collaborations that support shared ownership of all phases of community health improvement, including assessment, planning, investment, implementation, and evaluation.	1)	Focuses on research topics, questions, and outcomes most important to patients and those who care for them.
2)	Proactive, broad, and diverse community engagement to improve results.	2)	Works closely with a range of health care stakeholders— including patients, caregivers, scientists, clinicians, health systems, and insurers—to guide our research funding.
3)	A hospital's definition of community that encompasses both a significant enough area to allow for population-wide interventions and measurable results, and includes a targeted focus to address disparities among subpopulations.		
4)	Maximum transparency to improve community engagement and accountability.	3)	Requires that patients be engaged in the research we fund, not as subjects but as partners who help determine what to study and how.
5)	Use of evidence-based interventions and encouragement of innovative practices with thorough evaluation.		
6)	Evaluation to inform a continuous improvement process.		
7)	Use of the highest quality data pooled from, and shared among, diverse public and private sources.		

CHNA, Community Health Needs Assessment; PCORI, Patient-Centered Outcomes Research Institute.

## Discussion

The major aim of this study was to determine whether CHNA reports increase understanding of pediatric health priorities in Florida. Overall, results indicated that CHNAs do have the potential to identify and compare key health priorities across Florida by engaging community stakeholders and utilizing relevant databases. Across the 4 most populous areas in Florida, nonprofit hospitals unanimously endorsed access to health care and nutrition/exercise/obesity as the 2 most significant health challenges affecting pediatric patients.

Lack of access to health care as the top priority across hospitals was not completely unexpected. Florida is among 19 states that have not yet implemented the ACA's Medicaid expansion. As a result, Florida has the third largest number of nonelderly, uninsured individuals in the United States; this represents 2.4 million people, with 300,000 between the ages of 0 and 18 years.^10^ Apart from the shortage and affordability of health insurance, multiple barriers contribute to this problem, including difficulty locating a doctor and scheduling an appointment, long wait times, lack of transportation, inconvenient office hours, and cultural or language barriers.^11–13^ Future CHNA reports should include distinct estimates of these barriers with clear measures for improvement.

The second priority across all hospitals was nutrition/exercise/obesity. Childhood obesity rates in Florida have not improved over the last 5 years and have remained approximately 12%-13% for children between the ages of 2 and 18 years.^14^ Childhood obesity is associated with risk for high cholesterol, diabetes, high blood pressure, and many other health issues later in life.^15–17^ Unlike some other health priorities (eg, cancer), nutrition, exercise, and obesity may be addressed more effectively. However, it was noted that there was little consistency in how hospitals reported obesity-related issues. Some hospitals were very detailed in providing statistics on physical activity, nutrition, parents' perception of their child's weight, and actual rates of overweight and obesity in their patient population and community, whereas others included little quantitative or qualitative information in their report. A more detailed and standardized structure for inputting data into CHNAs would greatly improve their interpretability and the strength of the conclusions that can be drawn. Such improvements would enhance their utility for developing new interventions, identifying target populations, and setting clear goals to improve health outcomes. It also could be used to track changes in key health priorities over time in specific communities.

Moreover, it was noteworthy that hospitals in the Orlando and Miami regions had major discrepancies in their prioritization of health needs. These 2 regions exhibited the largest average difference in priority scores in comparison to any 2 metropolitan areas. Asthma/respiratory conditions were ranked as an extremely high priority in the Miami area, but were ranked at the bottom of the priority list for the Orlando region. Consequently, although state-level priorities may rank a particular illness or public health problem as critically important, a community may prioritize the same issue quite differently. These types of data, which reflect the unique, regional priorities of the community, can be used to tailor interventions more effectively to the specific needs of the local community—a potentially powerful use of this tool and the ultimate goal of CHNA reports. Further, the ability to better determine which regions and hospitals benefit most from particular interventions and treatments could serve as a pivotal asset for identifying PCORI's future research priorities.

CHNA analyses also revealed that hospitals across Florida that have distinct pediatric priorities clearly were more focused on addressing children's health concerns. These hospitals listed asthma/respiratory conditions, sexual health, dental care, immunizations, substance use disorders, and injury as critical health needs much more frequently than hospitals that combined adult and pediatric data. Conversely, hospitals that did not make this distinction mentioned illnesses and issues related primarily to adult or geriatric populations, such as cardiovascular disease, stroke, cancer, disparities, maternal health, and communicable diseases. Thus, the study team recommends separating pediatric health needs assessments from adults' data.

CHNA data that are reflective of pediatric priorities are critical for forming future partnerships and developing collaborative approaches to public health, by aligning hospitals that share common goals and foci. Pediatric hospitals should form or leverage existing consortia to develop consistent, standardized data collection strategies for CHNA reports to identify key health priorities for pediatric patients.

There are only 7 freestanding children's hospitals across the state of Florida. Therefore, only 7 CHNA reports covered pediatric care exclusively —the rest included a combination of pediatric, adult, and geriatric patients. As a result, the health needs prioritized in CHNA reports from those hospitals that care for multiple age groups are difficult to interpret and does not further an understanding of the true health needs of pediatric patients in Florida. Furthermore, the location of the freestanding children's hospitals only allow for an in-depth analysis of 4 communities (Miami-Dade, Jacksonville, Tampa, and Orlando metro areas). For these regions, the study team was able to derive a clear understanding of their health needs and priorities. Unfortunately, this is not possible for many other communities across Florida. Mandating that hospitals that provide pediatric services separate their data by pediatric versus adult needs in their CHNA report would greatly facilitate the analysis and interpretation of these data and provide a more accurate representation of the current health needs for both populations.

Equally important is the standardization of the format and protocol for ranking and prioritizing these community health needs. Each CHNA included in this study utilized its own methodology. Most of the reports simply stated the priorities without ordering them based on urgency, magnitude of the problem, or ability to affect change. The lack of structure and standardization of CHNAs diminished the interpretability of the results and made it challenging to derive meaningful information. These inconsistencies also made comparisons between CHNA reports very difficult. Despite the large investment of resources, time, and effort invested in producing these reports, they are difficult to use as guides for patient-centered research. Moving forward, the study team recommends that specific guidelines be established to standardize the methodological approach and variables included in CHNA reports.

One place to start would be to identify the inclusion of a standard set of domains with clear and appropriate definitions. The 18 priorities described in this study could serve as a guide. This would reduce the confusion associated with different terminologies and ensure that the same health issues are being evaluated by all hospitals. Second, the study team recommends a more granular decomposition of the 2 major priorities identified in this study (access to care, nutrition/exercise/obesity). Specifically, more detail is needed on insurance, physician availability, scheduling problems, wait times, lack of transportation, inconvenient office hours, and cultural or language barriers that reduce access to care. To better understand and address obesity, standardized data on physical activity, nutrition, parents' perception of their child's weight, and actual rates of overweight and obesity in the local community are needed to adequately target this complex health problem.

A third step in raising the level of data quality within CHNAs would be to require that hospitals rate their health needs on a set of scales based on urgency, magnitude of the problem, and ability to affect change. These ratings would allow for easier accessibility and interpretability of the health priorities documented in the CHNA report. These modest changes in the CHNA protocol, along with the separation of pediatric versus adult priorities, would ensure that these data are of high quality and produce compelling, clinically meaningful results. These changes also would make the linkages between CHNA reports and PCORI research priorities clearer and stronger.

## Conclusion

CHNA reports can be used to effectively identify community needs on both a regional and state level. The CHNA analyses in this study demonstrated that access to health care and nutrition/exercise/obesity are universally recognized by all Florida hospitals as key priorities for hospitals providing pediatric care in the 4 major metropolitan regions. However, the current methods, processes, and accessibility of these documents made them difficult to use as an informative pediatric research tool. Until more structure and standardization are implemented, it will be extremely difficult to use CHNA reports to generate goals for future research and intervention.

## Supplementary Material

Supplemental data

## Supplementary Material

Supplementary Table1
